# Morphological evidence suggestive of a hierarchical mode of glial cell diversification and intrinsic developmental plasticity within the murine enteric nervous system

**DOI:** 10.3389/fnins.2025.1701574

**Published:** 2025-12-03

**Authors:** Marie A. Lefèvre, Mylène Bourgeois, Rodolphe Soret, Nicolas Pilon

**Affiliations:** 1 Molecular Genetics of Development Laboratory, Département des Sciences Biologiques, Université du Québec à Montréal (UQAM), Montréal, QC, Canada; 2 Centre d’excellence en recherche sur les maladies orphelines – Fondation Courtois (CERMO-FC), Université du Québec à Montréal, Montréal, QC, Canada; 3 Département de pédiatrie, Université de Montréal, Montréal, QC, Canada

**Keywords:** adaptative compensation, developmental plasticity, enteric glial cells, enteric nervous system, enteric neurons, genetic cell lineage tracing, Hirschsprung disease, Schwann cell precursors

## Abstract

Enteric glial cells represent a highly heterogeneous cell population residing both within and outside the ganglia and inter-ganglionic fiber bundles that form the core networks of the enteric nervous system (ENS). Despite the availability of several relevant single-cell transcriptomic datasets, the classification of enteric glia in various subtypes is still mainly based on their particular location and associated morphological attributes. We recently reported that these “topo-morphological” enteric glia subtypes gradually appear during early postnatal development in healthy wildtype mice, under the influence of structural tissue changes that occur during this period. This process is also influenced by the source of ENS progenitors, with notable biased contributions by Schwann cell precursors (SCPs). This prior work further suggested the existence of a hierarchical program of spatiotemporal differentiation, whereby intra-network enteric glia (i.e., within ganglia and fiber bundles) of the myenteric plexus sequentially give rise to nearby extra-network enteric glia as well as to more distant intra- and extra-network enteric glia of the submucosal plexus. To learn more about enteric glia diversification, we reasoned that the *Nr2f1^Spt/Spt^* mouse model of Waardenburg syndrome type IV could be particularly informative, as the absence of ENS in the colon of these mice is specifically due to premature glial differentiation of ENS progenitors before birth. Our new analyses of the ENS-containing ileum from these mice now also reveal abnormal acquisition of enteric glia diversity after birth. These alterations of enteric glia diversification are in agreement with the previously proposed hierarchical differentiation model in wildtype mice. This work also uncovered an intriguing neuronal phenotype in *Nr2f1^Spt/Spt^* mice in which a reduction in the number of neurons is associated with an increase in their size. Many of these larger neurons also co-express the glia marker S100β, which together with the noted increased contribution of SCPs to the overall pool of enteric glia further highlight the remarkable plasticity of the developing ENS.

## Introduction

The enteric nervous system (ENS) is a specialized division of the peripheral nervous system that orchestrates gastrointestinal functions. It is mainly organized into two interconnected networks of ganglia, the myenteric and submucosal plexuses, which both contain enteric glial cells and neurons (Furness, 2012; Sharkey and Mawe, 2023). Unlike enteric neurons, many enteric glia can in addition be found outside of these core ganglionic networks (combining ganglia and inter-ganglionic fiber bundles). While the diversity of enteric neuron subtypes is getting well characterized both developmentally and functionally (Benthal et al., 2024; Drokhlyansky et al., 2020; May-Zhang et al., 2021; Morarach et al., 2021; Zeisel et al., 2018; Brehmer, 2021; Majd et al., 2025), our current understanding of enteric glia diversity is comparatively rather superficial (Drokhlyansky et al., 2020; Zeisel et al., 2018; Wright et al., 2021; Baghdadi et al., 2022; Guyer et al., 2023; Windster et al., 2025; Schneider et al., 2023; Scavuzzo et al., 2023). Yet, the crucial active roles collectively played by enteric glia in the regulation of gastrointestinal function in health and disease are now well recognized (Seguella and Gulbransen, 2021; Gonzales and Gulbransen, 2025; Lefèvre et al., 2023), underscoring the need to better understand their diversity. Single-cell sequencing techniques have identified up to nine enteric glia subpopulations in mice and eight in humans (Drokhlyansky et al., 2020; Zeisel et al., 2018; Wright et al., 2021; Baghdadi et al., 2022; Guyer et al., 2023; Windster et al., 2025; Schneider et al., 2023; Scavuzzo et al., 2023). However, the designation of ENS cell populations based on transcriptional signature is subjected to experimental biases and ultimately remains a suggestive exercise, explaining why there is still no consensus among all available studies (Majd et al., 2025). Hence, the most reliable classification system of enteric glia subpopulations still relies on topological and closely associated morphological characteristics that were first described more than 30 years ago (Hanani and Reichenbach, 1994). Four main subtypes are currently distinguished based on such topo-morphological criteria: highly branched Type I located in enteric ganglia, fibrous Type II cells in inter-ganglionic fiber bundles, unbranched multipolar Type III cells outside these core structures, and bipolar Type IV cells embedded in the circular and longitudinal muscle layers (Hanani and Reichenbach, 1994; Boesmans et al., 2015; Gulbransen and Sharkey, 2012; McCallum et al., 2020; Charrier and Pilon, 2017; Lefèvre et al., 2024).

In a recent study seeking to understand how these topo-morphological subtypes are generated in wild-type (WT) FVB mice, we discovered that enteric glia gradually diversify during the first 3 weeks after birth, when the ENS must adapt to significant structural changes in the maturing bowel wall (Lefèvre et al., 2024). Our detailed temporal analyses of both myenteric and submucosal plexuses in two spatially distant segments of the gastrointestinal tract (more structurally mature distal ileum *vs*. less mature distal colon) led us to propose a hierarchical model of topo-morphological diversification—which also considered prior reports indicating that the submucosal plexus initially forms via inward cell migration from the myenteric plexus (McKeown et al., 2001; Lasrado et al., 2017). The proposed model stipulated that co-occurring Type I and II enteric glia from the myenteric plexus sequentially give rise to nearby Types III and IV, as well as to enteric glia in the submucosal plexus, starting with Type II enteric glia which then also likely contribute to sequentially generate submucosal Types I and III. This prior work further revealed that the source of ENS progenitors may influence enteric glia diversity as well, as enteric glia can arise either from neural crest cells (NCCs) that directly colonize the developing gut or through an intermediary state of NCC-derived Schwann cell precursors (SCPs) that use extrinsic nerves as colonizing routes (Uesaka et al., 2015; Pilon, 2021). At the end of post-natal maturation at P20, we found that SCPs contribute to all topo-morphological subtypes, but not equally, preferentially contributing to Type IV enteric glia in the myenteric plexus/circular muscle and to Type II enteric glia in the submucosal plexus (Lefèvre et al., 2024).

Mouse models of developmental enteric neuropathies like Hirschsprung disease (aganglionic megacolon) can provide valuable insights into the basic mechanisms of ENS formation, including cell fate decisions. Notably, by focusing on the ENS-containing regions upstream of the aganglionic zone after birth, several groups have previously reported a common increased proportion of nitrergic neurons in several genetically distinct mutant mouse lines (Avila et al., 2025; Bhave et al., 2021; Bhave et al., 2022; Cheng et al., 2016; Musser et al., 2015; Roberts et al., 2008; Touré et al., 2019; Zaitoun et al., 2013). Interestingly, the SCP contribution to the overall pool of enteric neurons was also found to be specifically increased in the hypoganglionic transition zone preceding aganglionosis, mostly giving rise to the nitrergic subtype as well (Uesaka et al., 2021). However, only one study considered enteric glia in such analysis, reporting no significant differences in the overall proportions of enteric glia within the small intestinal and mid-colonic myenteric plexus of *Sox10^Dom/+^* mice (Musser et al., 2015). To the best of our knowledge, enteric glia diversification has never been investigated in this context. To begin addressing this question, we reasoned that mice homozygous for the *Spot* mutant allele of *Nr2f1* (*Nr2f1^Spt/Spt^*) would be especially well-suited. These mice phenocopy Waardenburg syndrome type IV (WS4), combining aganglionic megacolon with SCP/melanocyte-related abnormalities in the skin and inner ear (Bergeron et al., 2016; Bonnamour et al., 2022). The *Spot* mutation leads to neural crest-specific upregulation of the transcription factor NR2F1, which was found to abnormally reinforce the glial fate. In the developing ENS, this translates into premature enteric glia differentiation of rostro-caudally migrating NCCs, which thereby fail to fully colonize the colon (Bergeron et al., 2016). In SCPs, the overabundance of NR2F1 was found to rather prevent them from normally differentiating into melanoblasts (Bonnamour et al., 2022), with as yet undetermined consequences for the ENS.

In this study, we evaluated both enteric glia diversification and the contribution of SCPs in the postnatal distal ileum of *Nr2f1^Spt/Spt^* mice. Our immunofluorescence and genetic cell lineage tracing data align closely with the previously proposed hierarchical differentiation model, while also providing further evidence of the great plasticity of the developing ENS.

## Materials and methods

### Mice

Wild-type FVB mice [FVB/NCrl; Strain code 207] were purchased from Charles River Laboratory. *Spot* mice (*Nr2f1^Spt/+^*) were obtained through a pigmentation-based insertional mutagenesis screen aimed at identifying genes involved in NCC development (Pilon, 2016) and bred to homozygosity to generate diseased individuals. SCP-derived cells were detected using a Cre/loxP lineage tracing system. The *Dhh*-Cre line [Jax stock #012929; FVB*(Cg)-Tg(Dhh-cre)1Mejr/J*], obtained from the Jackson Laboratory, was crossed with the *Rosa26*^[*FloxedSTOP*]*YFP*^ line [*Gt(ROSA)26Sor^TM1(EYFP)Cos^*], kindly provided by Dr. Frank Costantini (Columbia University). All mouse lines were maintained on the FVB background and genotyped using either pigmentation pattern (for *Spot* mice) or standard PCR with primers listed in Supplementary Table 1. Breeding couples were housed in individually ventilated cages and fed the Charles River Rodent Diet #5075 (Cargill Animal Nutrition). All experiments were conducted in compliance with the guidelines of the Canadian Council on Animal Care (CCAC) and approved by the institutional committee (CIPA #992 and #959) of the Université du Québec à Montreal (UQAM). Euthanasia was performed by decapitation for mice aged between postnatal day (P) 1 to P5; or by carbon dioxide (CO_2_) exposure following isoflurane anesthesia for P15 mice. Animals of both sexes were included at each time point (see Supplementary Data Sheet 1).

### Sample collection and processing

Euthanized mice were pinned to a Styrofoam slab, and the skin and peritoneum were opened to access the gastrointestinal tract. The most distal centimeter of the ileum, located just upstream of the cecum, was dissected out in ice-cold 1X PBS (Phosphate-Buffered Saline). Each sample was cut longitudinally along the mesentery, then washed in 1X PBS to remove fecal material, and pinned with the mucosal side facing up onto Sylgard-coated (Dow Corning, Freeland, MI) Petri dishes. The pinned tissues were fixed overnight in 4% paraformaldehyde diluted in 1X PBS and washed three times for 10-min in 1X PBS the next day. Additional microdissection was performed on tissues from P15 mice, to strip away the mucosal layer (containing the submucosal plexus) from the muscle layers (including the myenteric plexus).

### Whole-mount immunofluorescence staining

Distal ileum samples were first permeabilized for 2 h in a blocking solution (5% Fetal Bovine Serum and 1% Triton-X100 in 1X PBS). They were then incubated overnight at 4 °C with primary antibodies diluted in the same blocking solution. Following three 10-min washes in 1X PBS, samples were incubated with relevant secondary antibodies, also diluted in blocking solution for 1 h 30. This step was followed by three additional washes of 10 min in 1X PBS. During the second wash, samples were counterstained with DAPI (4′,6-diamidino-2-phenylindole) diluted in 1X PBS. The list of antibodies and their respective dilution factors are provided in Supplementary Table 2. Finally, stained tissues were mounted between two 24 × 50 mm glass coverslips in 100% Glycerol, for plexus visualization by confocal microscopy.

### Imaging and data analysis

Immunofluorescence images were captured with a Nikon A1 confocal microscope equipped with Plan Fluor 20×/0.75 MImm and Plan Apo *λ* 60×/1.40 objectives. For each biological replicate, 3–10 representative z-stack projections of multiple 1 μm-thick images were acquired, and subsequently analyzed using the ImageJ software. As previously described (Lefèvre et al., 2024), we manually quantified SOX10+ or S100β+ enteric glia subtypes, using the “Cell Counter” plugin. We measured the surface area of ganglia, extraganglionic space, interganglionic fibers and neuronal cell bodies, using the “freehand selections” tool. As previously explained (Lefèvre et al., 2024), we excluded the longitudinal muscle layer from our analyses because of autofluorescence-related interference from the juxtaposed serosa.

### Statistics

Unless otherwise indicated, all experiments were conducted with 3 biological replicates per time point and condition. For each replicate, either 3–5 and/or 6–10 fields of view were analyzed in the myenteric plexus/circular muscle and submucosal plexus, respectively. Detailed information, including sex and the number of counted cells, is provided in Supplementary Data Sheet 1. Quantitative data are presented as mean ± standard deviation (SD), with the value of each imaging field represented by a single dot, except where otherwise indicated in the legend. Statistical comparisons between conditions were carried out in GraphPad Prism 9.5.1, using *t* test (for two groups) and One-Way or Two-Way ANOVA followed by Sidak’s multiple comparison tests (for more than two groups). Correlation analyses were performed by calculating the Pearson coefficient *r* and corresponding *p*-value. The statistical test applied is specified in each figure legend. Data were considered statistically significant when the *p*-value was less than 0.05.

## Results

### Emergence of enteric glia topo-morphological subtypes is altered in the ileal myenteric plexus of *Nr2f1^Spt/Spt^* mice

To evaluate whether and how enteric glia diversification could be perturbed in the context of a developmental enteric neuropathy, we first compared the emergence of enteric glia topo-morphological subtypes in the myenteric plexus and circular muscle of the distal ileum between WT (FVB) and *Nr2f1^Spt/Spt^* mice. As per our previously established methodology (Lefèvre et al., 2024), we performed whole-mount immunostaining for pan-glial (SOX10, S100β) and pan-neuronal (βIII-Tubulin) markers. We analyzed three developmental time points (P1, P5, P15) during the postnatal window of enteric glia diversification (Lefèvre et al., 2024), while also considering the early postnatal mortality of *Nr2f1^Spt/Spt^* pups (only ~28% survival at P20+) (Bergeron et al., 2016). As described in detail in our prior study (Lefèvre et al., 2024), quantification of relative subtype proportions was mainly based on the location of SOX10+ cells, while also using the gross morphology of S100β+ cells to help resolve ambiguities (e.g., Types I and II at the border of inter-ganglionic fibers). We specifically chose to study the distal ileum segment because ~70% of the colon is aganglionic in *Nr2f1^Spt/Spt^* mice; the transition zone is located in the proximal colon while the entire ileum is normally colonized by ENS progenitors (Bergeron et al., 2016).

**Figure 1:**
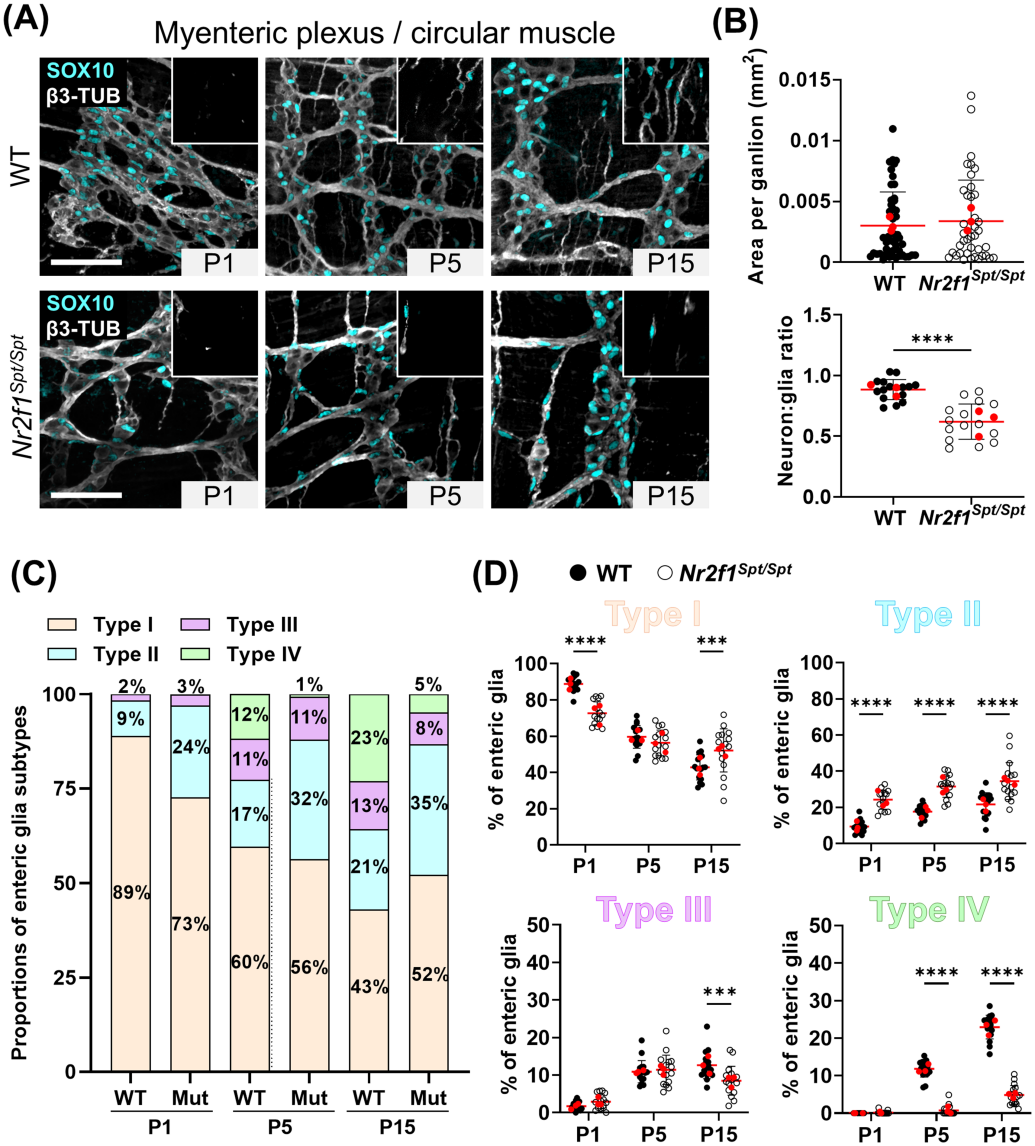
Analysis of enteric glia diversification in the postnatal myenteric plexus and circular muscle layer of the distal ileum from WT and *Nr2f1^Spt/Spt^* mice. **(A)** Immunofluorescence analysis of the myenteric plexus and associated circular muscle layer (insert at the top right) in the distal ileum from wild-type FVB and *Nr2f1^Spt/Spt^* mice, at indicated postnatal ages (P1, P5 and P15). Intestinal tissues were immunolabeled with antibodies against SOX10 for enteric glia (cyan) and βIII-Tubulin for neuronal fibers (gray). Displayed images are z-stack projections representative of observations made from *N* = 3 mice per time point. Scale bar, 70 μm. **(B)** Quantitative analysis of the area per ganglion (mm^2^, each dot represents a ganglion, *n* = 39–48 ganglia) and the neuron:glia ratio (total neurons:total Type I enteric glia, each dot represents a single 60× field of view; red dots indicate the average per animal) in the myenteric plexus of wild-type FVB and *Nr2f1^Spt/Spt^* mice at P15 (*N* = 3 mice; *n* = 5 60× fields of view). **(C,D)** Quantitative analysis of the relative proportions of enteric glia Types I to IV, in the myenteric plexus and the circular muscle layer of wild-type FVB (black dots) and *Nr2f1^Spt/Spt^* (white dots) mice, using images such as those displayed in panel A (*N* = 3 mice per time point; *n* = 5 60× fields of view per animal; red dots indicate the average per animal). **p ≤ 0.01, ****p ≤ 0.0001; *t* test **(B)**, Two-Way ANOVA and Šídák’s multiple comparison test **(D)**. Detailed information about each biological replicate, including sex and the number of counted cells, is also provided in Supplementary Data Sheet 1.

At first glance, myenteric ganglia segregation seems to be accelerated in the distal ileum of *Nr2f1^Spt/Spt^* mice at P1 and P5 (Figure 1A), although this does not affect the final average size of each ganglion at P15 (Figure 1B, top panel). However, the neuron:glia ratio is significantly lower in these ganglia at P15 (0.9 ± 0.1 in WT *vs.* 0.6 ± 0.2 in *Nr2f1^Spt/Spt^*) (Figure 1B, bottom panel), mostly because of a drastically reduced abundance of neurons compared to WT (823 ± 129 neurons/mm^2^ in WT *vs.* 462 ± 173 neurons/mm^2^ in *Nr2f1^Spt/Spt^*) (Supplementary Figure 1A). Intriguingly, the fact that ganglion size remains nonetheless unaffected in this context can be explained by a commensurate enlargement of these less numerous neurons in *Nr2f1^Spt/Spt^* mice, as we quantified based on anti-HuC/D staining (239 ± 50 μm^2^ in WT *vs.* 397 ± 124 μm^2^ in *Nr2f1^Spt/Spt^*) (Supplementary Figure 1B). It is also noteworthy that this increase in size is rather general (i.e., it does not affect only a few neurons) and is not accompanied by any morphological signs of degeneration such as deformation of the nucleus and/or vacuolation in the cytoplasm.

Important differences were also noted when analyzing enteric glia subtypes across the P1–P15 period. At P1, while ganglionic Type I cells constitute the majority of enteric glia in both WT and *Nr2f1^Spt/Spt^* mice, their proportion is significantly lower in the latter (89 ± 4% in WT *vs.* 73 ± 7% in *Nr2f1^Spt/Spt^*) (Figures 1A,C,D and Supplementary Figure 2A). This decrease is accompanied by a complementary increased proportion of Type II cells in fiber bundles (9 ± 4% in WT *vs*. 24 ± 6% in *Nr2f1^Spt/Spt^*) (Figures 1A,C,D and Supplementary Figure 2A). The low proportion of Type III cells remains unchanged (2–3 ± 1%), while Type IV cells are not present at this perinatal stage (Figures 1A,C,D and Supplementary Figures 2A, 3). At P5, WT mice exhibit an expansion phase for Type III and IV cells, with a concomitant reduction in Type I (Figures 1A,C,D and Supplementary Figures 2A, 3). Although a comparable increase in Type III is observed in *Nr2f1^Spt/Spt^* mice, Type IV emergence is markedly delayed (12 ± 2% in WT *vs.* 1 ± 1% in *Nr2f1^Spt/Spt^*), and Type II proportion remains elevated compared to WT (17 ± 4% in WT *vs.* 32 ± 6% in *Nr2f1^Spt/Spt^*) (Figures 1A,C,D and Supplementary Figures 2A, 3). At P15, *Nr2f1^Spt/Spt^* mice exhibit significant alterations in all four topo-morphological subtypes, with higher proportions of both Type I (43 ± 7% in WT *vs.* 52 ± 13% in *Nr2f1*^*Spt/*Spt^) and Type II (21 ± 6% in WT *vs.* 35 ± 11% in *Nr2f1^Spt/Spt^*) at the expense of Types III and IV (Figures 1A,C,D and Supplementary Figures 2A, 3). Although Type III proportions appear similar in both conditions before P15, a decline is now observed in *Nr2f1^Spt/Spt^* mice (13 ± 4% in WT *vs.* 8 ± 4% in *Nr2f1^Spt/Spt^*) (Figures 1A,C,D and Supplementary Figure 2A). Furthermore, the emergence of Type IV enteric glia is still robustly perturbed at this stage (23 ± 3% in WT *vs.* 5 ± 3% in *Nr2f1^Spt/Spt^*) (Figures 1A,C,D and Supplementary Figures 2A, 3).

**Figure 2:**
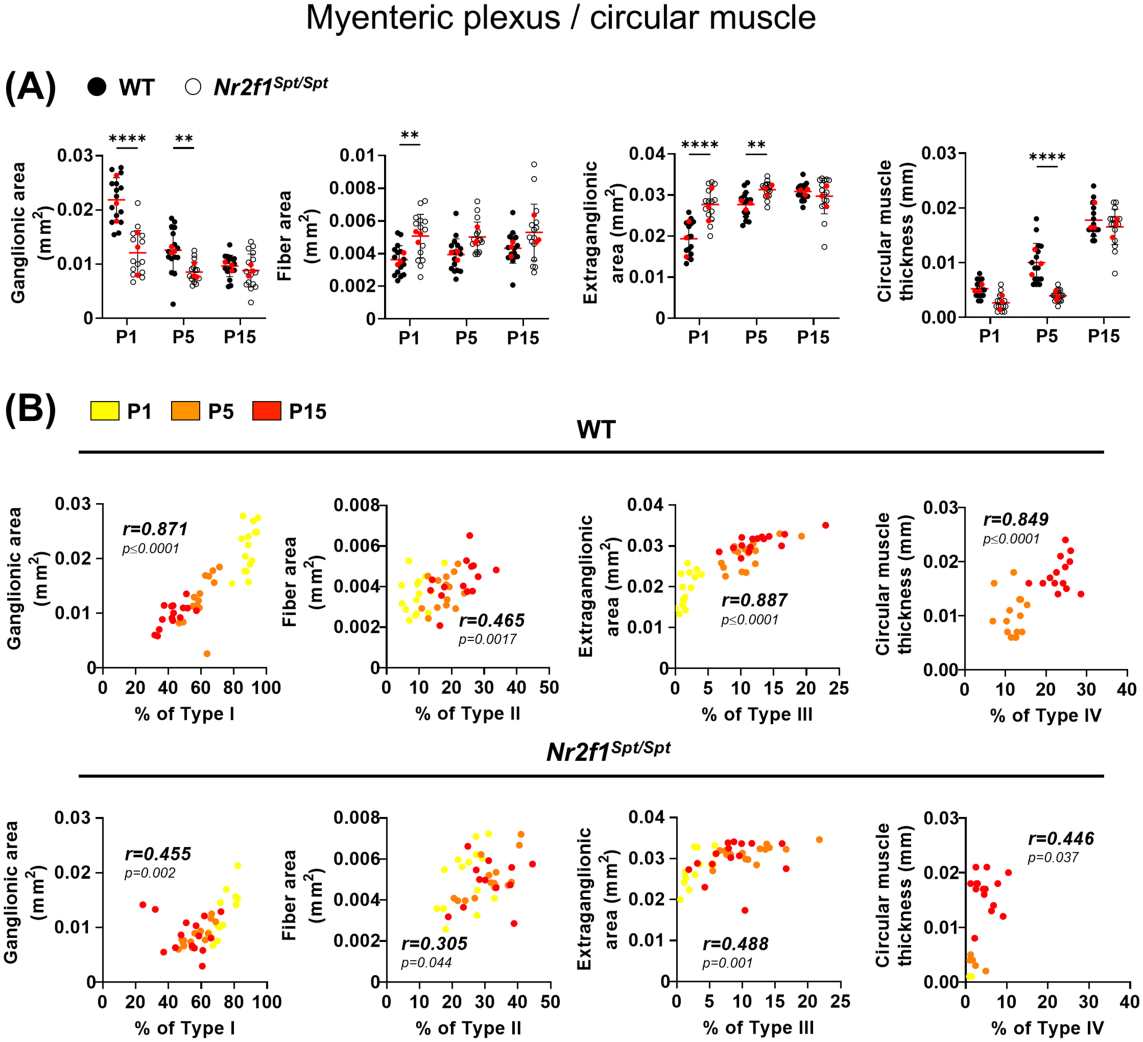
Analysis of structural maturation of the postnatal myenteric plexus and circular muscle layer in the distal ileum of WT and *Nr2f1^Spt/Spt^* mice. **(A)** Quantitative analysis of indicated morphometric parameters (ganglionic surface area, extraganglionic surface area, interganglionic fiber surface area and circular muscle thickness) in the distal ileum from wild-type FVB (black dots) and *Nr2f1^Spt/Spt^* (white dots) mice as a function of age during the early postnatal period (P1, P5, and P15; red dots indicate the average per animal). **(B)** Correlation analysis (excluding 0 and 100% values; incompatible with proportion calculations) between Type I proportion and ganglionic area, Type II proportion and interganglionic fiber area, Type III proportion and extraganglionic area, and Type IV proportion and circular muscle thickness, in wild-type FVB (top panels) and *Nr2f1^Spt/Spt^* (bottom panels) mice. Colored symbols correspond to the indicated time points. r, Pearson’s correlation coefficient. **p ≤ 0.01, ****p ≤ 0.0001; Two-Way ANOVA and Šídák’s multiple comparison test **(A)**.

Given that enteric glia diversification correlates with structural changes within the ENS and circular muscle layer during the early postnatal period (Lefèvre et al., 2024), we investigated whether the noted imbalance in enteric glia subtypes was accompanied by alterations of relevant morphometric parameters. From the same micrographs used to assess relative enteric glia proportions, we measured the overall ganglionic surface area, extra-ganglionic space, and inter-ganglionic fiber bundle thickness based on βIII-Tubulin expression in the myenteric plexus, as well as circular muscle thickness from immunofluorescence image stacks. Confirming our first impression that ganglia segregation was accelerated in *Nr2f1^Spt/Spt^* mice, we noted significant morphometric changes in the myenteric plexus at P1 and P5. Compared to WT mice, the surface area of both inter-ganglionic fiber bundles and extra-ganglionic space is increased and overall ganglionic area is reduced in *Nr2f1^Spt/Spt^* mice at these time points (Figures 1A, 2A). However, as previously noted when measuring average ganglion size (Figure 1B), no significant morphometric differences are observed between genotypes at P15 (Figures 1A, 2A), despite the continued imbalance in the relative proportions of Types I to III (Figures 1A,C,D). Additionally, thickening of circular muscle is delayed in *Nr2f1^Spt/Spt^* mice but is then also similar to WT by P15 (Figure 2A), even though Type IV deficits persist (Figures 1A,C,D and Supplementary Figures 2A, 3). In the end, for WT mice, strong correlations can again be observed between age-dependent changes in enteric glia subtype proportions and morphometric parameters during the P1–P15 period (Figure 2B, top panels), as we previously observed during the P1–P20 period (Lefèvre et al., 2024). However, this relationship is weaker in *Nr2f1^Spt/Spt^* mice, as indicated by lower Pearson correlation coefficients and reduced statistical significance across all correlation analyses (Figure 2B, bottom panels).

These findings show that in *Nr2f1^Spt/Spt^* mice, the overall temporal sequence of enteric glia diversification in the myenteric plexus and circular muscle layer is like in WT mice. However, the skewed subtype proportions suggest a partial defect in the transition from intra-network Types I and II to extra-network Types III and IV. These alterations coincide with perturbed structural maturation, in opposite directions, of the myenteric plexus (accelerated, with fewer but larger neurons in general) and the circular muscle layer (delayed).

### Emergence of enteric glia topo-morphological subtypes is altered in the ileal submucosal plexus of *Nr2f1^Spt/Spt^* mice

**Figure 3:**
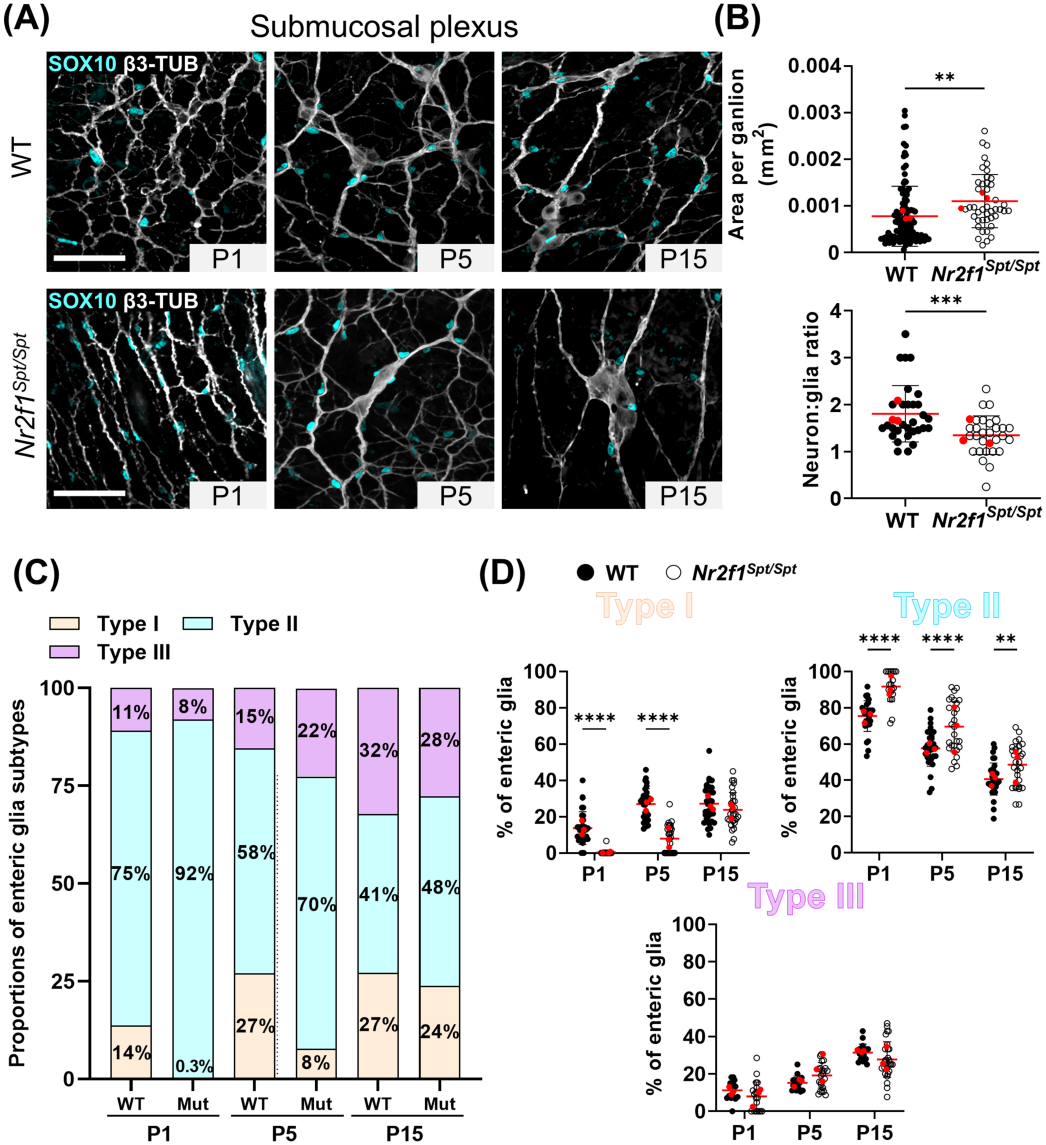
Analysis of enteric glia diversification in the postnatal submucosal plexus of the distal ileum from WT and *Nr2f1^Spt/Spt^* mice. **(A)** Immunofluorescence analysis of the submucosal plexus in the distal ileum from wild-type FVB and *Nr2f1^Spt/Spt^* mice, at indicated postnatal ages (P1, P5 and P15). Intestinal tissues were immunolabeled with antibodies against SOX10 for enteric glia (cyan) and βIII-Tubulin for neuronal fibers (gray). Displayed images are z-stack projections representative of observations made from *N* = 3 mice per time point. Scale bar, 70 μm. **(B)** Quantitative analysis of the area per ganglion (mm^2^, each dot represents a ganglia, *n* = 43–111) and the neuron:glia ratio (total neurons:total Type I enteric glia, each dot represents a single 60× field of view; red dots indicate the average per animal) in the myenteric plexus of wild-type FVB and *Nr2f1^Spt/Spt^* mice at P15 (*N* = 3 mice; *n* = 8–10 60× fields of view). **(C,D)** Quantitative analysis of the relative proportions of enteric glia Types I to IV, in the myenteric plexus/circular muscle layer of wild-type FVB (black dots) and *Nr2f1^Spt/Spt^* (white dots) mice, using images such as those displayed in panel A (*N* = 3 mice per time point; *n* = 8–10 60× fields of view per animal; red dots indicate the average per animal). *p ≤ 0.05, **p ≤ 0.01, ***p ≤ 0.001, ****p ≤ 0.0001; *t* test **(B)**, Two-Way ANOVA and Šídák’s multiple comparison test **(D)**. Detailed information about each biological replicate, including sex and the number of counted cells, is also provided in Supplementary Data Sheet 1.

We next applied the same experimental design to characterize potential alterations in the submucosal plexus of *Nr2f1^Spt/Spt^* mice. Here as well, we first noted that gangliogenesis is perturbed in these animals, but instead of an acceleration as in the myenteric plexus (Figure 1A), we observed a delay in the submucosal plexus (Figure 3A). Submucosal ganglia of *Nr2f1^Spt/Spt^* mice become overtly visible at P5 only (Figure 3A). Moreover, we found that these ganglia are less numerous and slightly larger in size compared to WT (Figure 3A), a persistent defect that we quantified at P15 (Figure 3B, top panel). As observed in the myenteric plexus (Figure 1B, bottom panel), the neuron:glia ratio is reduced in these submucosal ganglia at P15 (1.8 ± 0.6 in WT *vs.* 1.3 ± 0.4 in *Nr2f1^Spt/Spt^*) (Figure 3B, bottom panel), again mainly due to a lower than normal abundance of neurons (221 ± 113 neurons/mm^2^ in WT *vs.* 100 ± 49 neurons/mm^2^ in *Nr2f1^Spt/Spt^*) (Supplementary Figure 1C). Moreover, submucosal neurons are also larger in *Nr2f1^Spt/Spt^* mice compared to WT (185 ± 47 μm^2^ in WT *vs.* 365 ± 94 μm^2^ in *Nr2f1^Spt/Spt^*) (Supplementary Figure 1D).

As expected from the gangliogenesis defect described above, we observed a commensurate delay in the expansion of submucosal ganglionic Type I enteric glia in *Nr2f1^Spt/Spt^* mice between P1 and P5 (14 ± 9% at P1 to 27 ± 9% at P5 in WT *vs*. 0.3 ± 1% at P1 to 8 ± 8% at P5 in *Nr2f1^Spt/Spt^*), a delay that is later resorbed by P15 (Figures 3A,C,D and Supplementary Figure 2B). Fiber bundle-associated Type II cells are systematically the most abundant subtype in the submucosal plexus of WT mice before P20 (Lefèvre et al., 2024), regardless of the time-point investigated, and this is still the case in *Nr2f1^Spt/Spt^* mice (Figures 3A,C,D and Supplementary Figure 2B). Yet, while the relative proportion of Type II enteric glia was found to decrease throughout the analysis period, as Types I and III gradually appear, it is constantly higher in *Nr2f1^Spt/Spt^* mice compared to WT mice (75 ± 9% at P1 to 41 ± 9% at P15 in WT *vs*. 92 ± 9% at P1 to 48 ± 12% at P15 in *Nr2f1^Spt/Spt^*) (Figures 3A,C,D). However, no significant differences were observed for the expansion of Type III cell proportion between P1 and P15 (Figures 3A,C,D and Supplementary Figure 2B).

**Figure 4:**
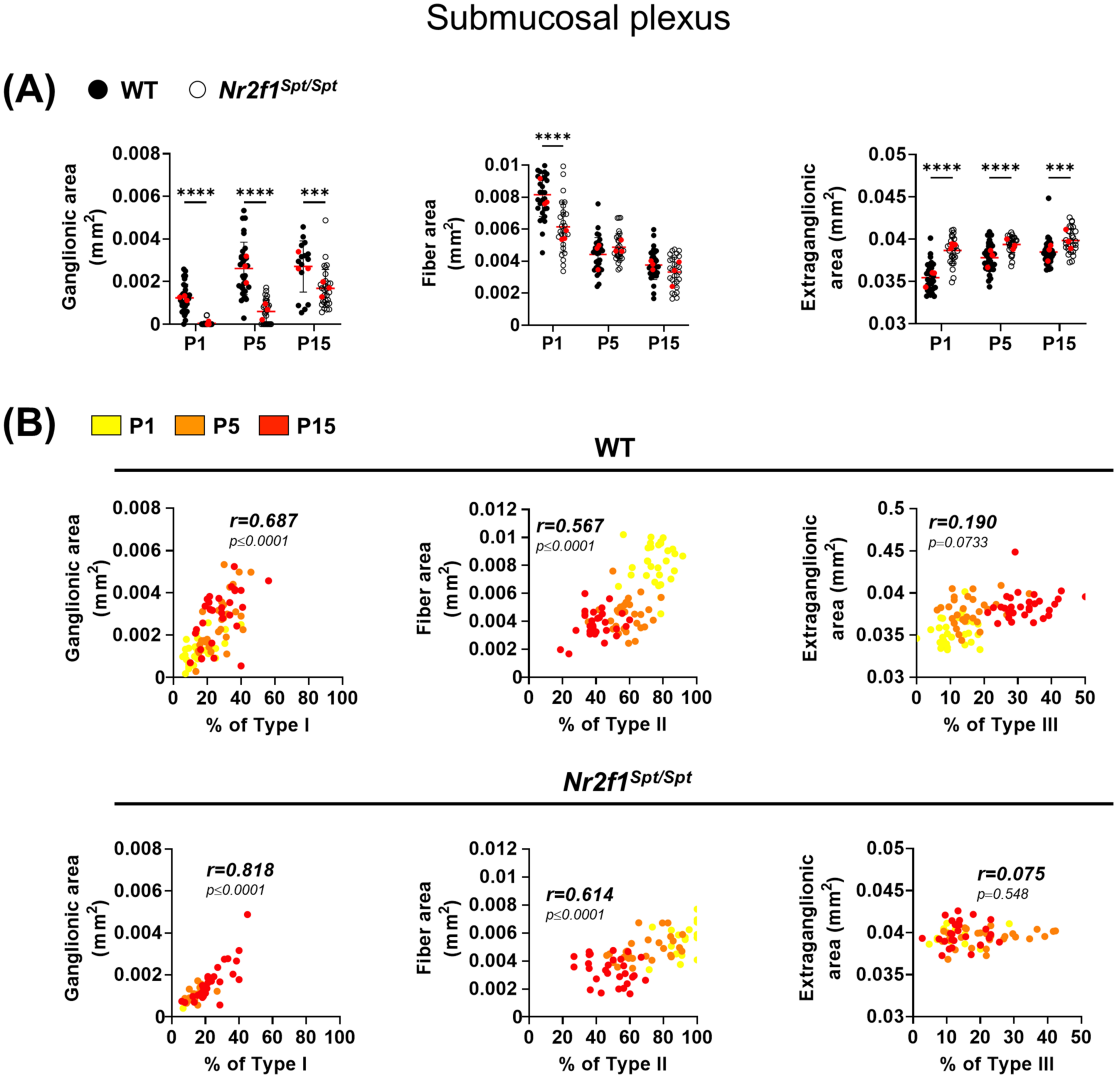
Analysis of structural maturation of the postnatal submucosal plexus in the distal ileum of WT and *Nr2f1^Spt/Spt^* mice. **(A)** Quantitative analysis of indicated morphometric parameters (ganglionic surface area, extraganglionic surface area and interganglionic fiber surface area) in the distal ileum from wild-type FVB (black dots) and *Nr2f1^Spt/Spt^* (white dots) mice as a function of age during the early postnatal period (P1, P5 and P15; red dots indicate the average per animal). **(B)** Correlation analysis (excluding 0 and 100% values; incompatible with proportion calculations) between Type I proportion and ganglionic area, Type II proportion and interganglionic fiber area, Type III proportion and extraganglionic area, in wild-type FVB (top panels) and *Nr2f1^Spt/Spt^* (bottom panels) mice. Colored symbols correspond to the indicated time points. r, Pearson’s correlation coefficient. **p ≤ 0.01, ***p ≤ 0.001, ****p ≤ 0.0001; Two-Way ANOVA and Šídák’s multiple comparison test **(A)**.

The larger size of individual submucosal ganglia in *Nr2f1^Spt/Spt^* mice (Figure 3B, top panel) most likely explains why the proportion of Type I enteric glia in these animals eventually reaches WT levels at P15 (Figures 3A,C,D), even though these ganglia are less numerous and hence overall ganglionic area is smaller from P1 to P15 (Figure 4A). Intriguingly, despite a higher proportion of Type II enteric glia in *Nr2f1^Spt/Spt^* mice compared to WT at every time point, fiber area is either slightly reduced (at P1) or undistinguishable from WT (at P5 and P15) (Figure 4A). Nonetheless, for both Type I and II enteric glia, correlations between relative subtype proportions and relevant morphometric parameters are as strong in *Nr2f1^Spt/Spt^* mice as they are in WT mice (Figure 4B). As expected, the altered gangliogenesis leads to a slightly increased extraganglionic area in *Nr2f1^Spt/Spt^* mice over the P1–P15 period (Figure 4A). However, we found no correlation between this morphometric parameter and the proportion of Type III enteric glia neither in *Nr2f1^Spt/Spt^* nor in WT mice (Figure 4B), in contrast to what we had previously observed in WT mice during the extended P1–P20 period (Lefèvre et al., 2024).

In sum, the accumulation of Type II cells and delayed expansion of Types I and III in *Nr2f1^Spt/Spt^* mice provide indirect evidence for the hypothesis that Type II enteric glia act as a local source for the other subtypes in the submucosal plexus. Accompanying neuronal changes in submucosal ganglia of *Nr2f1^Spt/Spt^* mice, again including fewer but larger healthy-looking neurons as observed in the myenteric plexus, further suggest the possibility of adaptive compensation to an initial delay in submucosal gangliogenesis. Such potential adaptative compensation might also contribute to maintaining strong correlations between the proportions of Type I and II enteric glia and their associated morphometric parameters in the submucosal plexus.

### Signs of post-natal adaptative compensation in the ileal ENS of *Nr2f1^Spt/Spt^* mice include a neuronal-like S100β+ cell population and an increased contribution by SCPs

**Figure 5:**
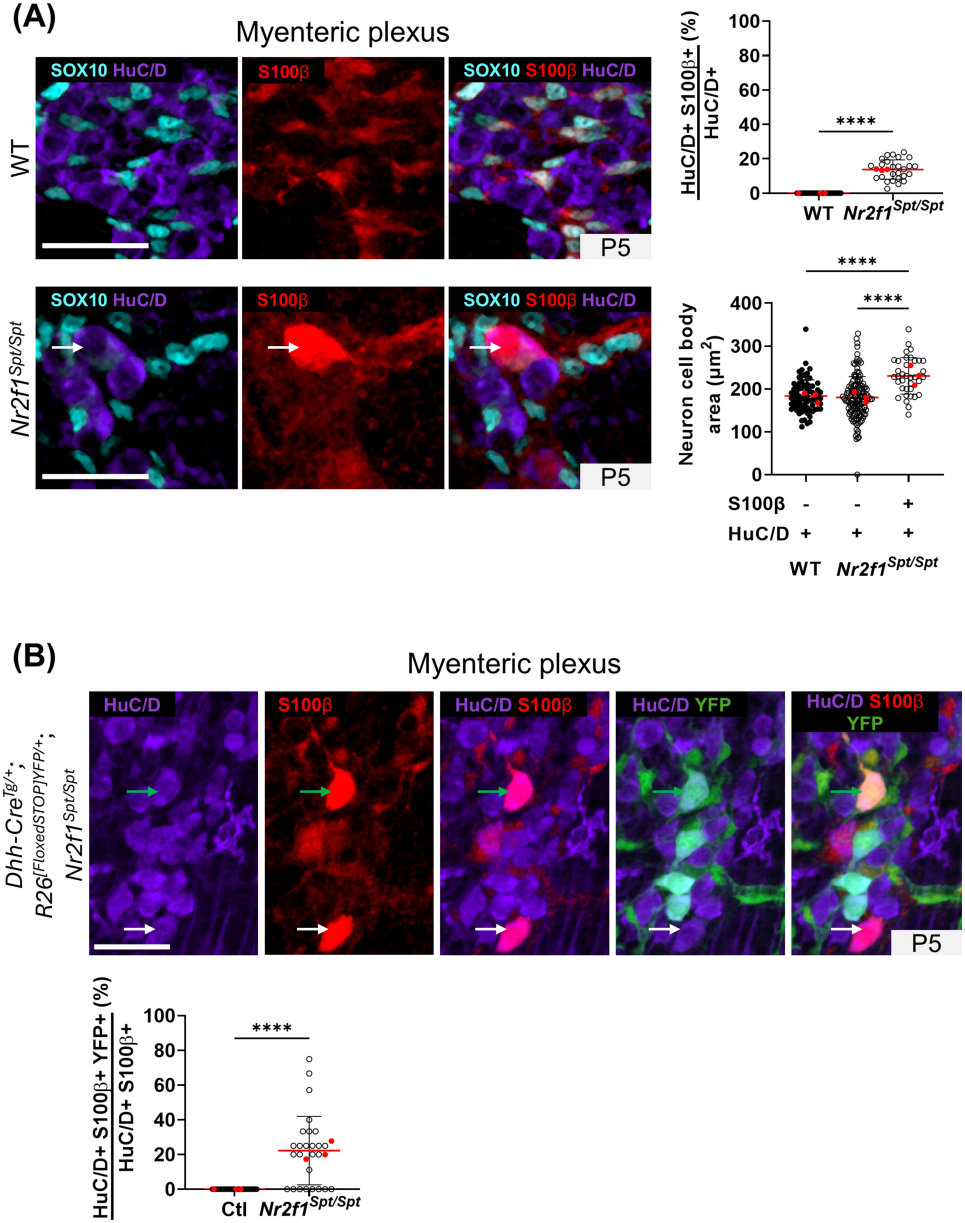
Analysis of the S100β+ HuC/D+ cell population in the myenteric plexus of the distal ileum from WT and *Nr2f1^Spt/Spt^* mice. **(A)** Immunofluorescence analysis of S100β expression pattern in the myenteric plexus of the distal ileum from P5 wild-type FVB and *Nr2f1^Spt/Spt^* mice (left panels), and accompanying quantitative analyses (right panels). Intestinal tissues were immunolabeled with antibodies against the pan-glial marker SOX10 (cyan), the pan-neuronal marker HuC/D (purple) and S100β (red). The white arrow points to a HuC/D and S100β double-positive cell. Displayed images are z-stack projections representative of observations made from *N* = 3 mice. Scale bar, 40 μm. The top right panel shows the quantitative analysis of HuC/D+ S100β+ proportion, where each dot represents the percentage of HuC/D+ S100β+ over the total of HuC/D + neurons in a single 60× field of view (*N* = 3 mice; *n* = 5 60× fields of view; red dots indicate the average per animal). Bottom right panel shows the quantitative analysis of cell body area of HuC/D + S100β+/− cells, where each dot represents a single cell body area (*N* = 3 mice; 5 60× fields of view per animal; *n* = 36–137 cell bodies measured in total; red dots indicate the average per animal). **(B)** Immunofluorescence analysis of the SCP contribution (YFP+) to HuC/D + S100β + cell population, in the myenteric plexus of the distal ileum from P5 *Nr2f1^Spt/Spt^* mice (left panels), and accompanying quantitative analysis (right panel). Intestinal tissues were immunolabeled with antibodies against the pan-neuronal marker HuC/D (purple), S100β (red) and GFP/YFP for SCP-derived cells (green). White arrows point to HuC/D and S100β double-positive cells. Displayed images are z-stack projections representative of observations made from *N* = 3 mice. Scale bar, 70 μm. The quantitative analysis shows the proportion of HuC/D + S100β + cells that are also positive for YFP in control *Dhh-Cre^Tg/+^*; *Rosa26^[FloxedSTOP]YFP/+^* and *Dhh-Cre^Tg/+^*; *Rosa26^[FloxedSTOP]YFP/+^*; *Nr2f1^Spt/Spt^* mice, with each dot representing the percentage of HuC/D+ S100β+ YFP+ over the total of HuC/D+ S100β+ cells in a single 60× field of view (*N* = 3 mice; *n* = 6–11 60× fields of view; red dots indicate the average per animal). ****p ≤ 0.0001; *t* test (% analyses), Two-Way ANOVA and Šídák’s multiple comparison test (Cell body area analysis). Detailed information about each biological replicate, including sex and the number of counted cells, is also provided in Supplementary Data Sheet 1.

While using anti-S100β immunofluorescence to clarify enteric glia subtype identity, we noticed that this glial marker was also often present in neuronal-like ganglion cells (i.e., with a large rounded nucleus typical of neurons) of *Nr2f1^Spt/Spt^* mice from P5 onwards, and most especially in the myenteric plexus (Supplementary Figure 4). In the distal ileum of WT mice at P5, S100β expression is normally restricted to enteric glia with SOX10+ nuclei, in both myenteric and submucosal ganglia (Figure 5 and Supplementary Figure 4). However, in the same region of age-matched *Nr2f1^Spt/Spt^* mice, several S100β+ ganglion cells do not stain positive for SOX10 (Supplementary Figure 4). As best evidenced in the myenteric plexus, the anti-S100β immunofluorescence signal is generally brighter in these SOX10-negative cells than in SOX10+ cells (Figure 5 and Supplementary Figure 4). Such bright S100β+ but SOX10-negative ganglion cells were rarely observed in WT mice, only in the submucosal plexus at the later P15 time-point (Supplementary Figure 4B).

As previously reported by others in different contexts (Grundmann et al., 2016; Parathan et al., 2020), S100β+ ganglion cells negative for SOX10 in *Nr2f1^Spt/Spt^* mice can be immunolabeled with the pan-neuronal marker HuC/D (Figure 5)—which is always absent from SOX10+ glia, even at the early P1 time point (Supplementary Figure 5). Our quantitative analyses in the myenteric plexus at P5, when and where these S100β+ HuC/D+ cells first appear in sufficient numbers, indicate that they constitute 14 ± 6% of total HuC/D+ neurons (Figure 5A). We also found that myenteric S100β+ HuC/D+ neuronal-like cells are slightly larger in size compared to neighbor HuC/D+ neurons that are negative for S100β, or to those of WT mice (Figure 5A), suggesting a preferential contribution to the global increase in neuron size previously noted at P15 (Supplementary Figures 1B,D).

To evaluate the possibility that these S100β+ HuC/D+ neuronal-like cells could be specifically derived from SCPs, we turned to a genetic cell lineage tracing approach based on the *Dhh-Cre* driver (Jaegle et al., 2003) that we and others have successfully used in prior studies (Uesaka et al., 2015; Bonnamour et al., 2022; Soret et al., 2020; Bonnamour et al., 2021). As we previously validated in the developing ENS before birth (Lefèvre et al., 2024), when *Dhh-Cre* is used in conjunction with a Cre reporter allele like *Rosa26^[FloxedSTOP]YFP^* (Srinivas et al., 2001), it allows to specifically label SCPs on extrinsic nerves and none of the intrinsic ENS cells derived directly from NCCs. Our analysis of *Dhh-Cre^Tg/+^*; *Rosa26^[FloxedSTOP]YFP/+^*; *Nr2f1^Spt/Spt^* triple-transgenics at the same P5 stage as above revealed a heterogeneous contribution of SCPs to the pool of S100β+ HuC/D+ neuronal-like cells, with high variability among myenteric ganglia (Figure 5B). With 22 ± 21% of S100β+ HuC/D+ neuronal-like cells also positive for YFP in the *Nr2f1^Spt/Spt^* background, SCPs do contribute but clearly not exclusively (Figure 5B).

**Figure 6:**
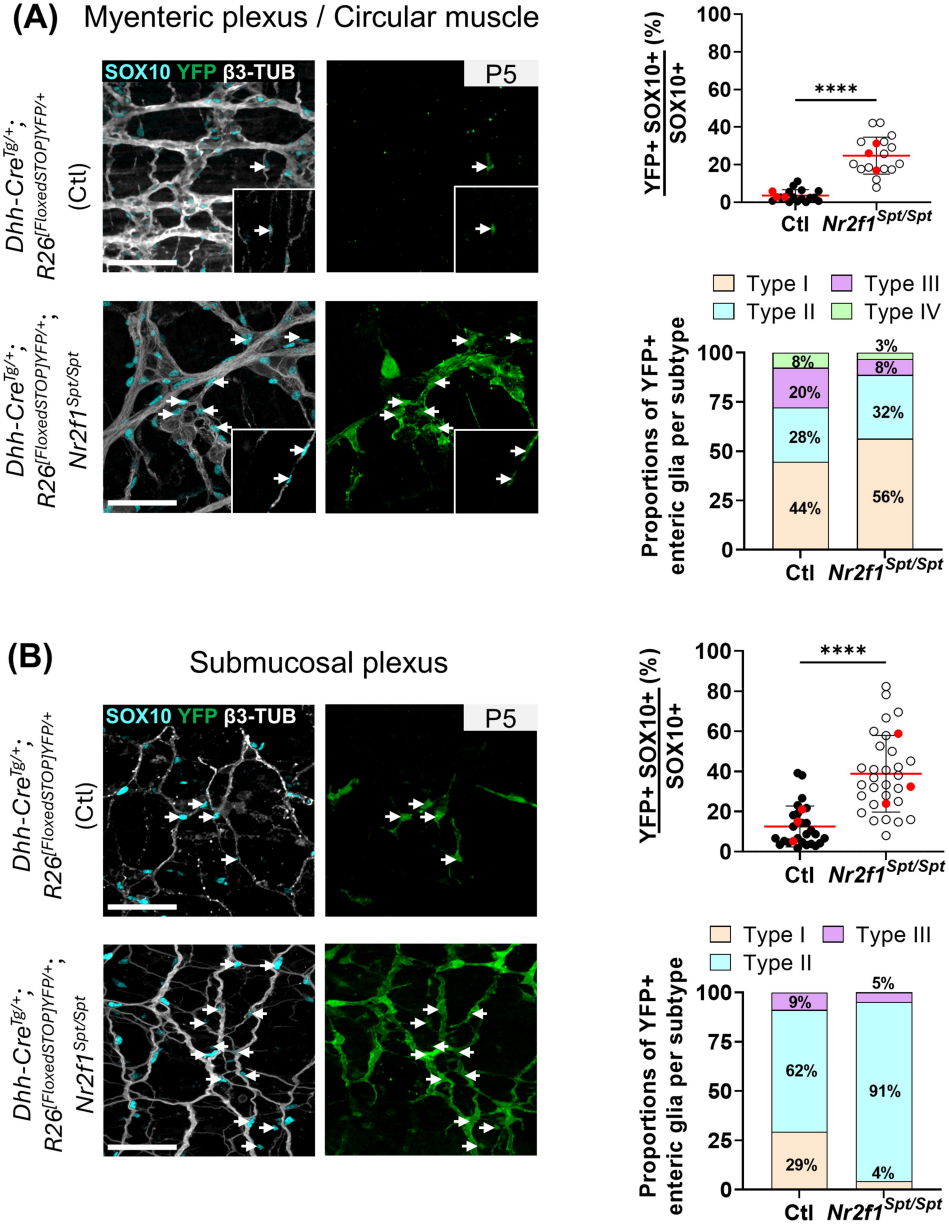
Analysis of the SCP contribution to enteric glia diversification in the distal ileum of control and *Nr2f1^Spt/Spt^* mice. **(A,B)** Immunofluorescence analysis of the SCP contribution (YFP+) to enteric glia diversification in the myenteric plexus/circular muscle layer **(A)** and the submucosal plexus **(B)** of the distal ileum from P5 control *Dhh-Cre^Tg/+^; R26^[FloxedSTOP]YFP/+^* (Ctl) and *Dhh-Cre^Tg/+^; R26^[FloxedSTOP]YFP/+^; Nr2f1^Spt/Spt^* mice, and accompanying quantitative analyses. Intestinal tissues were immunolabeled with antibodies against SOX10 for enteric glia (cyan), GFP/YFP for SCP-derived cells (green) and βIII-Tubulin for neuronal fibers (gray). White arrows point to YFP + cells derived from SCPs. Displayed images are z-stack projections representative of observations made from *N* = 3 mice. Scale bar, 70 μm. Quantitative analyses show the SCP contribution to the global pool of enteric glia (percentage of YFP+ SOX10+ enteric glia among all SOX10+ enteric glia, top panels) and the SCP contribution per enteric glia subtype (bottom panels) (*N* = 3 mice; *n* = 5 fields of view per tissue for the myenteric plexus/muscular layer; *n* = 10 fields of view per tissue for the submucosal layer; red dots indicate the average per animal). ****p ≤ 0.0001; *t* test. Detailed information about each biological replicate, including sex and the number of counted cells, is also provided in Supplementary Data Sheet 1.

Importantly, the same cell lineage tracing approach also allowed us to evaluate the overall SCP contribution to enteric glia diversification in the *Nr2f1^Spt/Spt^* background. Once again, we limited these analyses to the P5 stage—which, in any case, is almost impossible to go beyond considering the low yield of multi-allele crosses that now added to the high death rate (~72%) of *Nr2f1^Spt/Spt^* animals before weaning age (Bergeron et al., 2016). In both plexuses, we observed robust increases of the SCP contribution to the overall pool of enteric glia (Figure 6), with respective variations peaking at ~6× in the myenteric plexus (4 ± 4% in controls *vs.* 25 ± 11% in *Nr2f1^Spt/Spt^* background, Figure 6A) and ~3× in the submucosal plexus (13 ± 11% in controls *vs.* 39 ± 20% in *Nr2f1^Spt/Spt^* background, Figure 6B). In each case, the global increase is not equally distributed among the different enteric glia subtypes (Figure 6 and Supplementary Figure 6), mostly aligning with the skewed proportions already noted at P5 in favor of Types I/II in the myenteric plexus and Type II in the submucosal plexus (Figures 1C, 3C).

All these data are consistent with the notion that multiple compensatory mechanisms can be spontaneously turned on in the early postnatal ENS to help adapt and/or circumvent developmental anomalies. In the distal ileum of *Nr2f1^Spt/Spt^* mice, this includes an atypical contribution by S100β+ HuC/D+ neuronal-like cells and a globally increased contribution of SCPs.

## Discussion

For the current study, we hypothesized that the *Nr2f1^Spt/Spt^* mouse model of Waardenburg syndrome Type IV (Pilon, 2021; Bergeron et al., 2016; Bonnamour et al., 2022), in which enteric gliogenesis is prematurely engaged during prenatal development (Charrier and Pilon, 2017; Bergeron et al., 2016), could also provide useful insights into the diversification process of enteric glia during the early postnatal period (P1–P15). We specifically selected the distal ileum from these mice as a region of interest to analyze the spatiotemporal acquisition of enteric glia subtype diversity in both the myenteric and submucosal plexuses. This specific bowel segment was chosen to compare with our previous analysis of enteric glia diversification in pre-weaned WT mice (Lefèvre et al., 2024)—which included both the distal ileum and distal colon—despite extensive colonic aganglionosis in *Nr2f1^Spt/Spt^* mice placing the hypoganglionic transition zone in the proximal colon (Bergeron et al., 2016). Still based on our prior analysis of WT mice (Lefèvre et al., 2024), we also considered the structural changes that occur during the early postnatal period as well as the contribution of SCPs to each enteric glia subtype. As discussed in more detail below, our new data in *Nr2f1^Spt/Spt^* mice corroborate the potential hierarchical mode of spatiotemporal enteric glia diversification previously inferred from our analysis of WT mice, while also highlighting the great developmental plasticity of the immature ENS after birth.

The hierarchical mode of enteric glia diversification that we previously proposed based on detailed spatiotemporal data from WT mice stipulated that intra-network Type I/II enteric glia from the myenteric plexus constitute the source of extra-network Type III and IV enteric glia, as well as Type II enteric glia in the submucosal plexus which then also contribute to submucosal Type I and III enteric glia (Lefèvre et al., 2024). As we were hoping, similar spatiotemporal analysis of enteric glia diversification in *Nr2f1^Spt/Spt^* mice proved to be informative in that it lends more credence to this hypothetical model—which nonetheless remains to be formally tested via experimentally challenging cell lineage tracing and imaging approaches, as we previously discussed (Lefèvre et al., 2024). Indeed, in both plexuses of these mutant mice, the expansion of “secondary” enteric glia subtypes (i.e., myenteric Types III/IV and submucosal Types I/III) at the expense of “primary” enteric glia subtypes (i.e., myenteric Types I/II and submucosal Type II) is significantly delayed compared to WT mice. Morphometric parameters previously suggested as influencing acquisition of enteric glia diversity also remain largely correlated with their respective associated enteric glia subtype in *Nr2f1^Spt/Spt^* mice, although such correlations are weaker in the myenteric plexus. In contrast, the contribution of SCPs to enteric glia diversification was found to differ from control mice, instead aligning with the skewed proportions in favor of “primary” enteric glia in both plexuses. These data for SCP-derived enteric glia in *Nr2f1^Spt/Spt^* mice are nonetheless in agreement with the general idea that SCPs can somehow sense and adapt to the pre-existing composition of the ENS, as previously reported for enteric neurons (Uesaka et al., 2021).

Unexpectedly, although the distal ileum of *Nr2f1^Spt/Spt^* mice appears to be fully colonized by neural crest-derived ENS progenitors before birth (Bergeron et al., 2016), gangliogenesis was found to be significantly altered in both plexuses. While the noted defects were initially found to differ in a temporal manner (accelerated for myenteric ganglia *vs*. delayed for submucosal ganglia), we then noted striking similarities in the accompanying neuronal abnormalities. In both ganglionic networks, markedly reduced neuron numbers are associated with a commensurate increase in the average size of individual neurons, so that the overall ganglionic surface area in *Nr2f1^Spt/Spt^* mice eventually becomes similar (in the myenteric plexus) or close (in the submucosal plexus) to WT levels at the latest investigated time-point (P15). The decreased neuronal abundance makes sense considering that gliogenesis is favored during prenatal ENS formation in *Nr2f1^Spt/Spt^* mice (Bergeron et al., 2016). The fact that these less abundant neurons are larger is much more surprising. To the best of our knowledge, this has not been reported in any context other than aging (Nguyen et al., 2023). While aging-associated hypertrophy of enteric neurons is believed to negatively alter their function (Nguyen et al., 2023), it is tempting to speculate that this is the converse in the developmental context of *Nr2f1^Spt/Spt^* mice. Indeed, in contrast to aging neurons in which the increase in size is accompanied by dysmorphic features, the morphology of enlarged neurons in *Nr2f1^Spt/Spt^* mice is overtly normal. Hence, if accompanied by a proportional increase of relevant subcellular and/or molecular attributes, larger enteric neurons could perhaps process more information more efficiently, as previously suggested in the CNS (Goriounova et al., 2018). Hence, having such “enhanced” neurons could be a potential adaptative way for the ENS to prevent functional impairment despite lower neuronal density. On the contrary, if the observed larger enteric neurons instead prove to be less efficient than normal, this would add to neuronal subtype imbalance (Avila et al., 2025; Bhave et al., 2021; Bhave et al., 2022; Cheng et al., 2016; Musser et al., 2015; Roberts et al., 2008; Touré et al., 2019; Zaitoun et al., 2013) as potential reason to explain why the ENS-containing segment fails to fully restore motility after surgical removal of the aganglionic segment in Hirschsprung disease. Clearly, this unexpected discovery raises a lot of neuron-oriented questions for future functional studies, far beyond the scope of the current study focused on enteric glia diversification.

Regardless of the mechanism(s) responsible for the aforementioned neuronal defect, the consequence of a decrease in the number of neurons might well be the reason for the alteration in the cascade of enteric glia diversification from the myenteric plexus. The reduced abundance of myenteric neurons could somehow force ganglionic Type I enteric glia with neurogenic potential (Guyer et al., 2023; Mueller et al., 2024) to replenish the neuronal pool within myenteric ganglia instead of contributing to extra-ganglionic Type III and IV enteric glia. This hypothesis is supported by our own observations of HuC/D+ neuronal-like cells also positive for the enteric glia marker S100β in these ganglia as well as by other studies suggesting that Type I enteric glia are the most neurogenic enteric glia subtype (Mueller et al., 2024). Interestingly, the presence of HuC/D+ S100β+ neuronal-like cells has also been observed during normal early postnatal ENS development in WT mice (Grundmann et al., 2016; Parathan et al., 2020), most especially in the small intestine but excluding the distal ileum (Grundmann et al., 2016). This suggests that enteric glia-derived neurogenesis is an inherent property of the developing ENS soon after birth that, if needed, can spontaneously be activated in bowel segments not necessarily prone to it normally. Moreover, studies in mouse models of ulcerative colitis (Belkind-Gerson et al., 2017) and Chagas disease (Khan et al., 2024) even suggest that this potential is maintained later in life and may manifest itself during the remission phase of these diseases.

The reduced abundance of neurons in the distal ileum of *Nr2f1^Spt/Spt^* mice is also likely the reason for the increased contribution of SCPs to the general pool of enteric glia. A similar result has been previously reported for SCP-derived neurons in the hypoganglionic transition zone of *Sox10^Venus/+^* and *Ednrb^−/−^* mouse models of Hirschsprung disease, just upstream of the aganglionic segment (Uesaka et al., 2021). The increased contribution by SCPs in these models was exclusively detected in the hypoganglionic region, being otherwise very low in the upstream ganglionated (neuron-rich) region or downstream aganglionic (neuron-devoid) one (Uesaka et al., 2021). Although the distal ileum of *Nr2f1^Spt/Spt^* mice is not hypoganglionic *per se* from a structural point of view, it does contain fewer neurons than normal and hence likely corresponds to the transition zone in the other genetic models mentioned above. Collectively, these observations suggest that reduced neuron density, but not their absence, is really the trigger of the increased SCP contribution to the ENS, independently of the underlying genetic defects. To further verify this hypothesis, we also analyzed neuronal density, neuron:glia ratio and SCP contribution in the distal ileum of heterozygous *Nr2f1^Spt/+^* mice. Strikingly, this analysis revealed an increased contribution of SCPs to the enteric glia pool specifically in the submucosal plexus where there are fewer neurons than normal, and not in the myenteric plexus where neuronal density is similar to WT mice (Supplementary Figure 7).

In conclusion, our work positions the distal ileum of *Nr2f1^Spt/Spt^* mice as an interesting playground not only to better understand enteric glia diversification but also to investigate potential mechanisms of adaptative compensation and developmental plasticity within the ENS. While the notion of a hierarchical mode of enteric glia diversification under the influence of structural changes has been strengthened, our findings show that the SCP contribution is more flexible than initially thought, being adaptable as a function of the tissue environment (where neuron density is a primary factor). Future work along these lines will certainly be helpful to better understand and/or improve ENS-regenerative therapies that are currently being developed based on either transplantation or *in situ* stimulation of enteric glia and SCPs (Soret et al., 2020; Mueller et al., 2024; Gary et al., 2025; Pan et al., 2022).

## Data Availability

The original contributions presented in the study are included in the article/Supplementary material, further inquiries can be directed to the corresponding author.
