# Supplementary material for: Morphological evidence suggestive of a hierarchical mode of glial cell diversification and intrinsic developmental plasticity within the murine enteric nervous system
Source: Front Neurosci. 2025 Dec 3;19:1701574. doi: 10.3389/fnins.2025.1701574 (PMC12708518; doi:10.3389/fnins.2025.1701574)
Supplement: Supplementary file 2 [file Supplementary_file_1.docx]

Supplementary Material

# Supplementary Figures

**Figure S1. Analysis of cell density and neuronal cell body area in myenteric and submucosal ganglia of the distal ileum from WT and *Nr2f1^Spt/Spt^* mice at P15.**

**Figure S2. Detailed cell count of enteric glia subtypes in myenteric and submucosal plexuses of the distal ileum from WT and *Nr2f1^Spt/Spt^* mice.**

**Figure S3. Immunofluorescence analysis of Type IV emergence in the circular muscle layer of the distal ileum from WT and *Nr2f1^Spt/Spt^* mice.**

**Figure S4. S100β expression pattern in myenteric and submucosal plexuses of the distal ileum from WT and *Nr2f1^Spt/Spt^* mice.**

**Figure S5.** **Representative images of SOX10 and HuC/D expression pattern in the distal ileum of WT and *Nr2f1^Spt/Spt^* mice at P1.**

**Figure S6.** C**ontribution of SCPs to topo-morphological enteric glia** **subtypes, detailed per sample.**

**Figure S7. Comparative analysis of the SCP contribution and neuronal density in the distal ileum from control, *Nr2f1^Spt/+^* and *Nr2f1^Spt/Spt^* mice.**

**
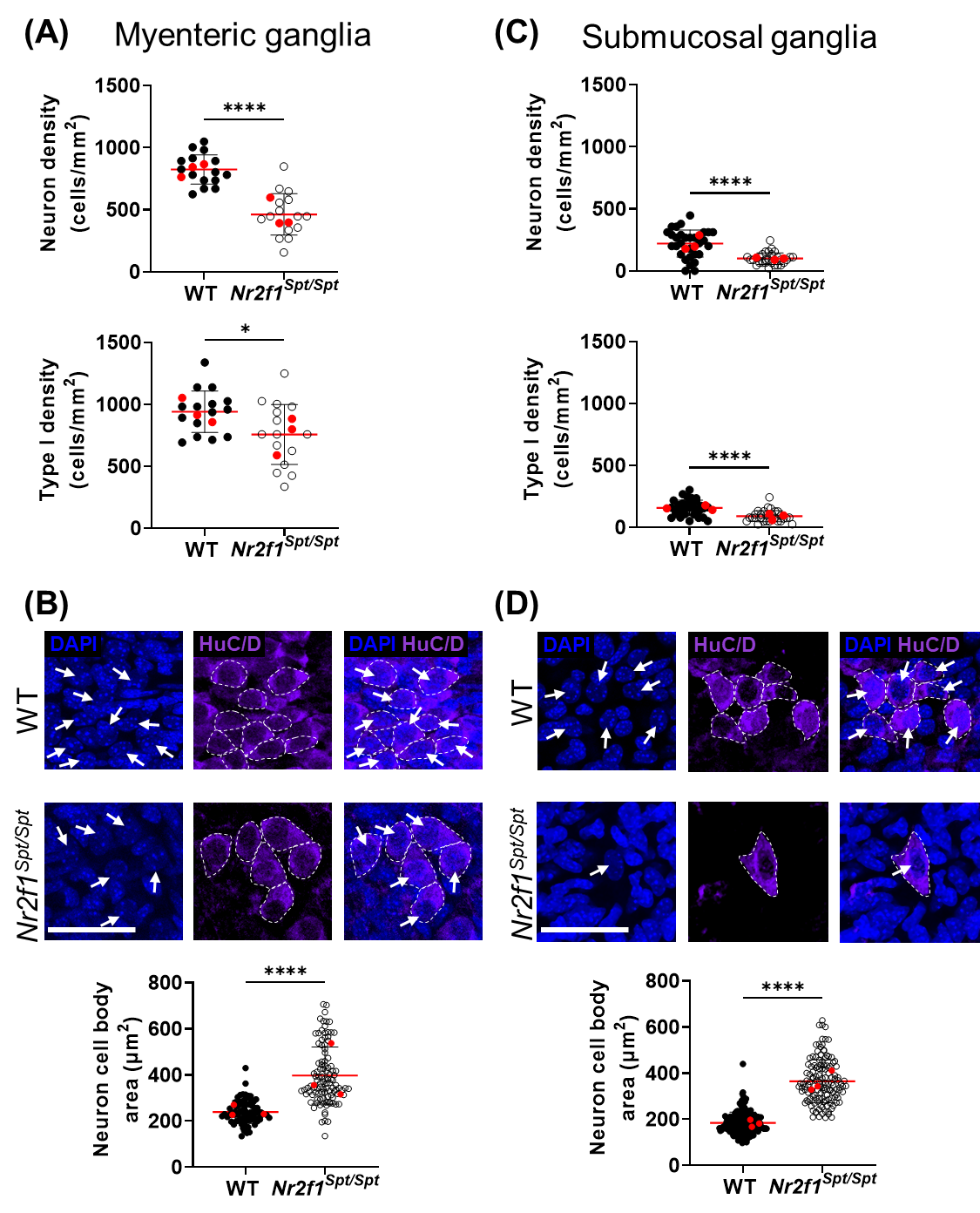
**

**Figure S1. Analysis of cell density and neuronal cell body area in myenteric and submucosal ganglia of the distal ileum from WT and *Nr2f1^Spt/Spt^* mice at P15.**

(**A, C**) Quantitative analysis of neuronal and Type I enteric glia density in myenteric (**A**) and submucosal (**C**) ganglia of the distal ileum from wild-type FVB and *Nr2f1^Spt/Spt^* mice (N=3 mice per time point; n=5-10 60x fields of view per animal; red dots indicate the average per animal). **(B, D**) Immunofluorescence analysis of myenteric (**B**) and submucosal (**D**) HuC/D+ neurons in the distal ileum from wild-type FVB and *Nr2f1^Spt/Spt^* mice, and accompanying quantitative analysis of cell body area delineated by dotted lines. White arrows point to DAPI-stained nuclei. Displayed images are z-stack projections representative of observations made from N=3 mice. Scale bar, 40μm. Each dot of the quantitative analysis represents a single cell body area (N=3 mice; 4-10 60X fields of view per animal; n=74-174 cell body measured in total; red dots indicate the average per animal). **P*≤0.05, *****P*≤0.0001; *t* test.

**
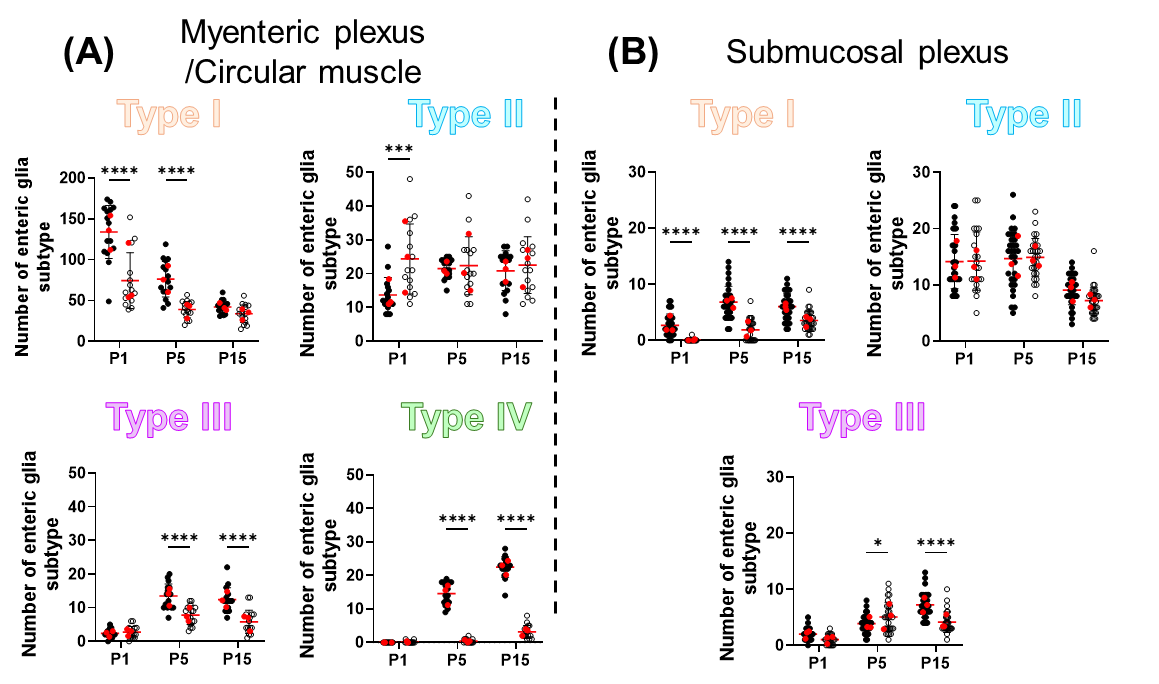
**

**Figure S2. Detailed cell count of enteric glia subtypes in myenteric and submucosal plexuses of the distal ileum from WT and *Nr2f1^Spt/Spt^* mice.**

(**A, B**) Quantitative analysis of the number of enteric glia subtypes per immunofluorescence micrograph of the myenteric plexus / circular muscle (A; Types I to IV) and the submucosal plexus (B; (Types I to III) of wild-type FVB (black dots) and *Nr2f1^Spt/Spt^* (white dots) mice, using images such as those displayed in Figures 1, 3 and S3 (N=3 mice per time point; n=5-10 60x fields of view per animal; each dot represents a field of view; red dots indicate the average per animal). ***P*≤0.01, *****P*≤0.0001; Two-Way ANOVA and Šídák's multiple comparison test.

**
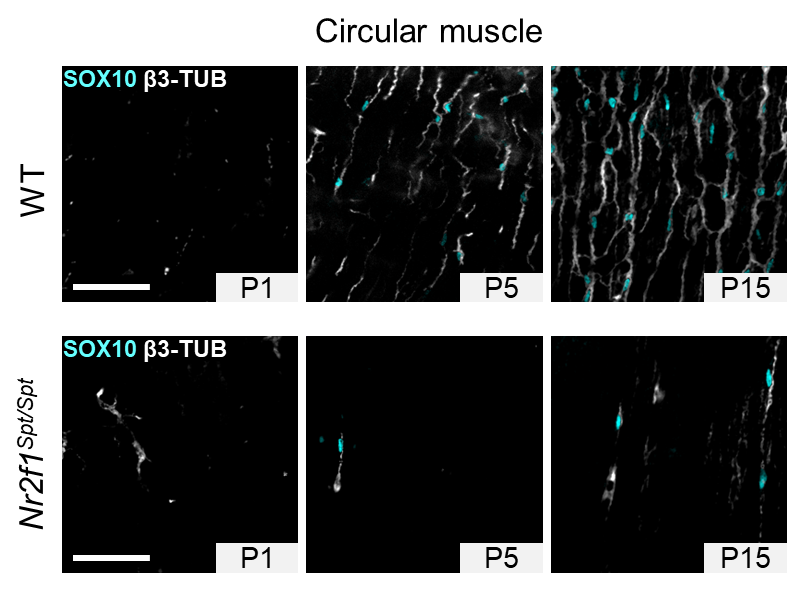
**

**Figure S3. Immunofluorescence analysis of Type IV emergence in the circular muscle layer of the distal ileum from WT and *Nr2f1^Spt/Spt^* mice.**

Representative images of the circular muscle layer of the distal ileum from wild-type FVB and *Nr2f1^Spt/Spt^* mice, at indicated postnatal ages (P1, P5 and P15). Intestinal tissues were immunolabeled with antibodies against SOX10 for enteric glia (cyan) and βIII-Tubulin for neuronal fibers (grey). Displayed images are z-stack projections representative of observations made from N=3 mice per time point. Scale bar, 70μm.

**
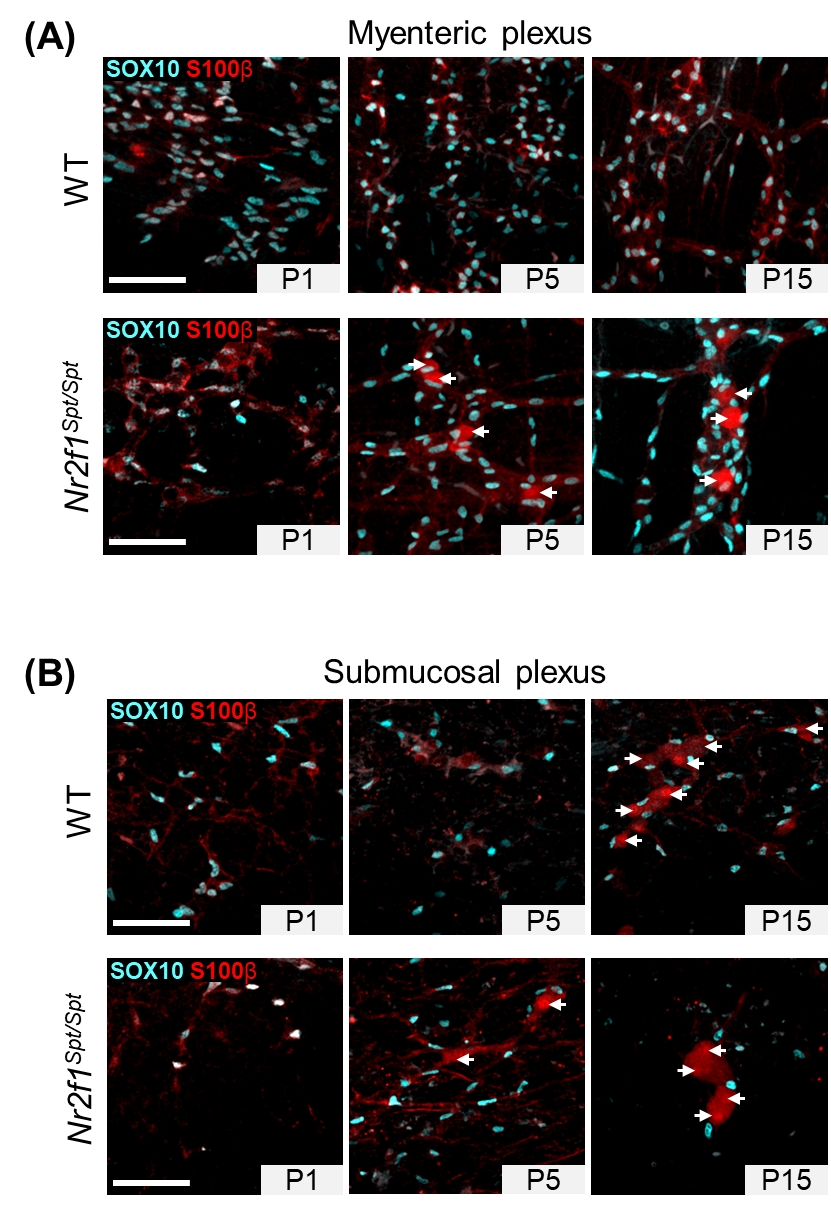
**

**Figure S4. S100β expression pattern in myenteric and submucosal plexuses of the distal ileum from WT and *Nr2f1^Spt/Spt^* mice.**

(**A-B**) Representative images showing the expression pattern of the glial marker S100β in myenteric (**A**) and submucosal (**B**) plexuses of the distal ileum from wild-type FVB and *Nr2f1^Spt/Spt^* mice, at indicated postnatal ages (P1, P5 and P15). Intestinal tissues were immunolabeled with antibodies against SOX10 (cyan) and S100β (red). White arrows point to cells with a neuron-like morphology that express S100β. Displayed images are z-stack projections representative of observations made from N=3 mice per time point. Scale bar, 70μm.

**
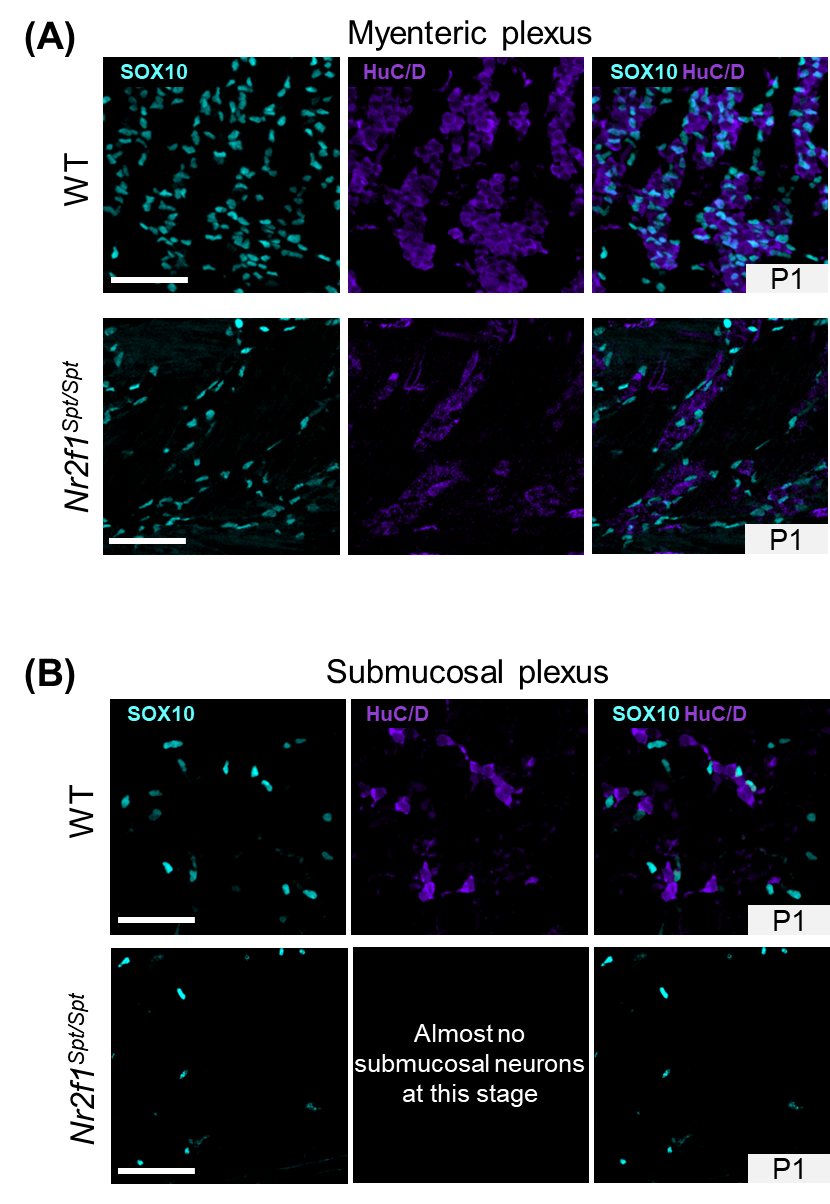
**

**Figure S5. Representative images of SOX10 and HuC/D expression pattern in the distal ileum of WT and *Nr2f1^Spt/Spt^* mice at P1.**

(**A, B**) Immunofluorescence analysis of the myenteric plexus (**A**) and the submucosal plexus (**B)** in the distal ileum from wild-type FVB and *Nr2f1^Spt/Spt^* newborns. Intestinal tissues were immunolabeled with antibodies against SOX10 for enteric glia (cyan) and HuC/D for neuronal cell body (purple). SOX10 and HuC/D staining do not overlap, indicating that SOX10 expression is restricted to enteric glia. Displayed images are z-stack projections representative of observations made from N=3 mice. Scale bar, 70μm.

**
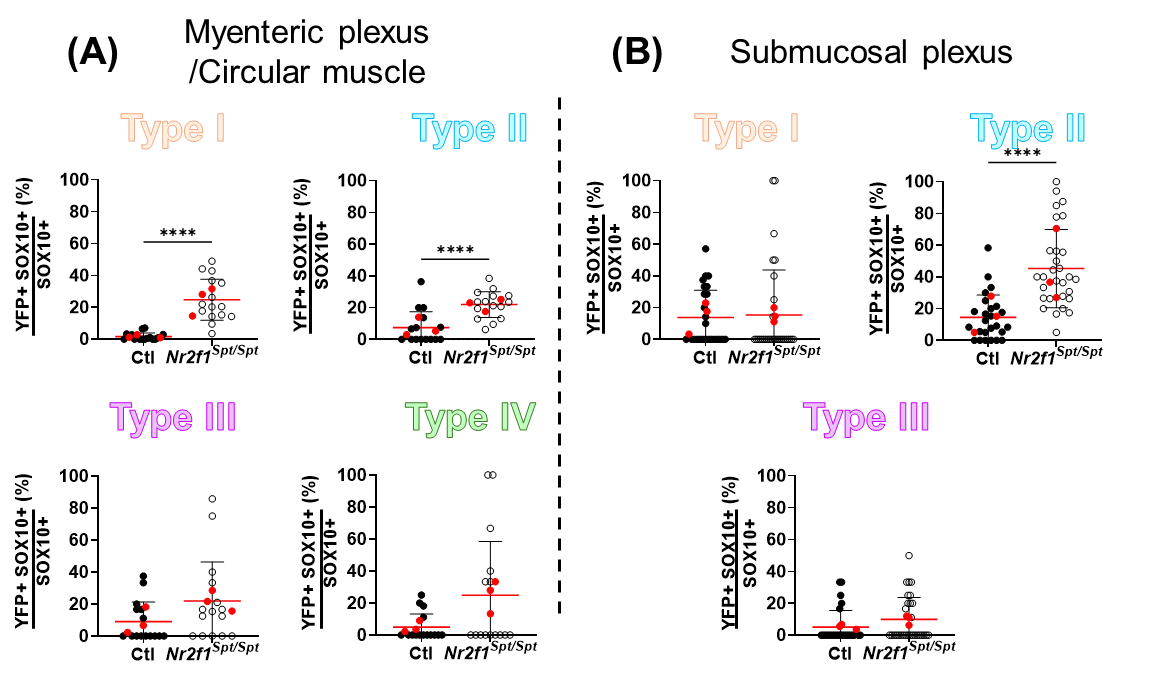
**

**Figure S6.** C**ontribution of SCPs to topo-morphological enteric glia** **subtypes, detailed per sample.**

(**A-B)** Quantitative analysis of the proportion of YFP+ SOX10+ enteric glia among total SOX10+ Type I, II, III or IV enteric glia in the myenteric plexus/circular muscle (**A**) and the submucosal plexus (**B**) of the distal ileum from P5 control *Dhh-Cre^Tg/+^;R26^[FloxedSTOP]YFP/+^* (Ctl) and *Dhh-Cre^Tg/+^; R26^[FloxedSTOP]YFP/+^;Nr2f1^Spt/Spt^* mice**.** Each dot represents the percentage of YFP+ SOX10+ enteric glia Types I, II, III or IV in a single 60X field of view (N=3 mice per time point, n=5 fields of view per tissue for the myenteric plexus/muscular layer; n= 6-10 fields of view per tissue for the submucosal layer; each dot represents a field of view; red dots indicate the average per animal). ****P*≤0.001, *****P*≤0.0001; *t*-test.

**
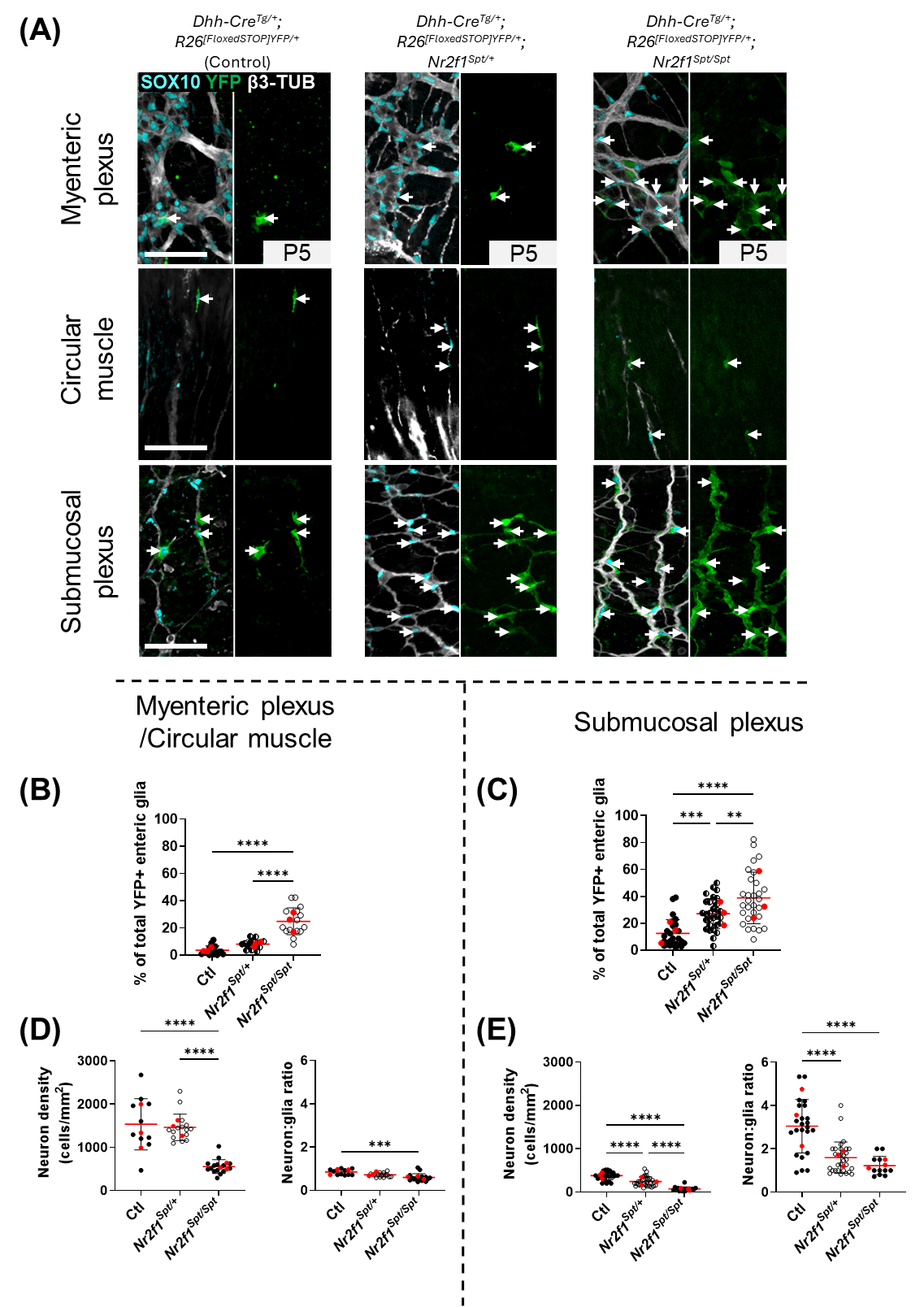
**

**Figure S7. Comparative analysis of the SCP contribution and neuronal density in the distal ileum from control, *Nr2f1^Spt/+^* and *Nr2f1^Spt/Spt^* mice.**

(**A**) Immunofluorescence analysis of the SCP contribution (YFP+) to the global pool of enteric glia in the myenteric plexus / circular muscle layer and the submucosal plexus of the distal ileum from P5 control *Dhh-Cre^Tg/+^; R26^[FloxedSTOP]YFP/+^* (Ctl), *Dhh-Cre^Tg/+^; R26^[FloxedSTOP]YFP/+^;Nr2f1^Spt/+^* and *Dhh-Cre^Tg/+^; R26^[FloxedSTOP]YFP/+^;Nr2f1^Spt/Spt^* mice. Intestinal tissues were immunolabeled with antibodies against SOX10 for enteric glia (cyan), GFP/YFP (green) and βIII-Tubulin for neuronal fibers (grey). White arrows point to YFP+ cells derived from SCPs. Displayed images are z-stack projections representative of observations made from N=3 mice per time point. Scale bar, 70μm. (**B-C**) Quantitative analysis of the SCP contribution to the global pool of enteric glia (percentage of YFP+ SOX10+ enteric glia among all SOX10+ enteric glia) in the myenteric plexus/circular muscle layer (**B**) and the submucosal plexus (**C**). **(D-E)** Quantitative analysis of neuron density and neuron:glia ratio in the myenteric plexus/circular muscle layer **(D)** and the submucosal plexus **(E)** of the distal ileum from control (black dots), *Nr2f1^Spt/+^*(half white half black dots), *Nr2f1^Spt/Spt^* (white dots) mice (N=3 mice per time point; n=6-10 60x fields of view per animal; red dots indicate the average per animal). ***P*≤0.01, ****P*≤0.001, *****P*≤0.0001; One-Way ANOVA and Šídák's multiple comparison test.

# Supplementary Tables

**Table S1. Oligonucleotide primers used for genotyping.**

| **GENE** | **SENSE PRIMER** | **ANTISENSE PRIMER** |
| --- | --- | --- |
| ***Rosa26-YFP*** | 5’CCCAAAGTCGCTCTGAGTTGTTATC3’ | YFP: 5’TGCGCCCTACAGATCCCTTAATTAA3’  WT: 5’CCAGATGACTACCTATCCTCCCA3’ |
| ***Dhh-Cre*** | 5’GATGAGGTTCGCAAGAACCTGATG3’ | 5’AACAGCATTGCTGTCACTTGGTCG3’ |
| ***Nr2f1^Spt/+^*** | 5’TTTGTGGCTGTAAAGATTGACTCC3’ | Mutant: 5’TTTAGCAAATCCCAGTCATTTCTTAG3’ WT: 5’TTAGGTCCTGGAGTCTGGTTAGTT3’ |

**Table S2. Primary and secondary antibodies used for immunofluorescence.**

| **ANTIBODY** | **SOURCE** | **HOST SPECIES** | **DILUTION** |
| --- | --- | --- | --- |
| **SOX10** | R&D Systems, #AF2864 | Goat | 1:500 |
| **S100β** | Novus Biologicals, #NBP1-41373SS | Rabbit | 1:500 |
| **βIII-Tubulin** | Abcam, #ab78078 | Mouse | 1:500 |
| **Green Fluorescent Protein (GFP)** | Abcam, #ab290 | Rabbit | 1:500 |
| **Green Fluorescent Protein (GFP)** | Abcam, #ab5450 | Goat | 1:500 |
| **HuC/HuD** | ThermoFisher, #A-21271 | Mouse | 1:500 |
| **AlexaFluor 594 Anti-rabbit** | Jackson ImmunoResearch Laboratories Inc, 711-585-152 | Donkey | 1:500 |
| **AlexaFluor 647 Anti-mouse** | Jackson ImmunoResearch Laboratories Inc, 715-605-150 | Donkey | 1:500 |
| **AlexaFluor 488 Anti-goat** | Jackson ImmunoResearch Laboratories Inc, 805-545-180 | Bovine | 1:500 |
